# Patients’ and Health Care Providers’ Evaluation of Quality of Life Issues in Advanced Cancer Using Functional Assessment of Chronic Illness Therapy - Palliative Care Module (FACIT-Pal) Scale

**DOI:** 10.4021/wjon578w

**Published:** 2012-10-28

**Authors:** Luluel Khan, Liang Zeng, David Cella, Nemica Thavarajah, Emily Chen, Liying Zhang, Margaret Bennett, Kenneth Peckham, Sandra De Costa, Jennifer L. Beaumont, May Tsao, Cyril Danjoux, Elizabeth Barnes, Arjun Sahgal, Edward Chow

**Affiliations:** a Department of Radiation Oncology, Odette Cancer Centre, University of Toronto, Toronto, Ontario, Canada; b Department of Medical Social Sciences, Northwestern University Feinberg School of Medicine, Chicago, IL, USA

**Keywords:** Advanced cancer patients, Quality of life, FACIT-pal, Health care professional

## Abstract

**Background:**

To examine the agreement of Health Care Providers (HCPs) and patients’ evaluation of quality of life on the Functional Assessment of Chronic Illness therapy - Palliative care module (FACIT-Pal) scale.

**Methods:**

Sixty advanced cancer patients and fifty-six health care providers involved in their care at Sunnybrook Health Sciences Centre completed a modified version of the FACIT- Pal. In the survey, patients and HCPs indicated the 10 top issues affecting the quality of life of patients with advanced cancer most profoundly. The percentage of participants selecting each item as one of their 10 most relevant items was calculated in HCPs and patients.

**Results:**

There were differences in relative rankings of QOL issues among patients and HCPs. Among the top 10 items which were identified from both patients and HCPs, there were differences in the rankings. Patients ranked emotional support from family (40.9%) as most important followed by pain (38.6%), lack of energy (31.8%) and able to enjoy life (29.6%). HCPs ranked in the following order: pain (73.2%), lack of energy (63.4%), nausea (51.2%) and dyspnea (51.2%) whereas patients rated nausea at 18.2 % and dyspnea at 9.09%.

**Conclusion:**

There is a discrepancy between scores of patients and HCPs as they may prioritize differently. HCPs tended to put more emphasis on physical symptoms, whereas patients had emotional and global issues as priorities.

## Introduction

Patients with advanced cancer are often poly-symptomatic due to the disease itself or as a result of treatments they receive. Therefore, in these patients especially, symptom control and quality of life (QOL) become more appropriate endpoints to measure, over more traditional endpoints such as survival. The assessment of QOL requires accurate and reliable instruments; various tools have been utilized to understand the needs of advanced cancer patients.

Patients with metastatic cancer often experience their own distinct symptoms and emotional issues when facing advanced cancer and its treatment. The patient’s QOL is affected by many other factors; including limited mobility, reduced performance, treatment side-effects and impaired role functioning. In patients with significantly limited functional ability, it may be necessary for family members or their caretakers to complete QOL assessments. In previous studies by our group and others, it has become evident that health care providers (HCPs) and patients may prioritize their concerns differently [1] and therefore, such proxy assessment may not be reliable.

The FACIT-Pal [2] is a combination of the FACT-G [3] plus a palliative specific subscale that was designed for use in patients in palliative care. The purpose of this study was therefore to compare the relative important of issues as rated by patients and HCPs.

## Methods

Sixty patients with advanced cancer and 56 health-care professionals (HCPs) involved in their care at Sunnybrook Health Sciences Centre, Toronto, Canada, evaluated all items of the FACIT-Pal on relevance and relative importance. Patient demographics were summarized as mean, standard deviation (SD), median, inter-quartiles, and ranges for age and KPS; proportions for gender, primary cancer site, clinic and patient status. HCP demographics were also summarized by years of professional experience, gender and profession.

Both patients and HCPs ranked the top ten most relevant and important issues. Patients were asked to consider the relevance and importance of each item to their current treatments and care, whereas HCPs were asked to answer based on their experience with palliative patients in general, not focusing on specific cases. The percentage of participants selecting each item as one of their 10 most relevant items was calculated in HCPs and patients. This study was approved by the Research Ethics Board at Sunnybrook Health Sciences Centre. All analyses were calculated by Statistical Analysis Software (SAS version 9.2 for Windows).

## Results

The FACIT-Pal (Table 1) was presented to a total of 60 patients (Table 2) and 56 HCPs who participated in this study (Table 3). Mean age of patients was 66 years, median KPS was 70, and the majority of patient participants were male (62%). Primary cancers of the prostate (33%), breast (18%) and lung (12%) were most common. Most patients had metastases to the bone, were enrolled from a radiation oncology clinic and were outpatients. The other patients had either brain or lung metastases. HCPs included in this analysis had on average 7 years of experience in their current field. The majority was radiation oncologists (43%), followed by radiation therapists (18%) and nurses (11%); genders were balanced (male: 55%).

**Table 1:** Items Included in the FACIT-Pal and Their Item Codes

Physical Well-being	GP1	I have a lack of energy
	GP2	I have nausea
	GP3	Because of my physical condition, I have trouble meeting the needs of my family
	GP4	I have pain
	GP5	I am bothered by side effects of treatment
	GP6	I feel ill
	GP7	I am forced to spend time in bed
Social/Family Well-being	GS1	I feel close to my friends
	GS2	I get emotional support from my family
	GS3	I get support from my friends
	GS4	My family has accepted my illness
	GS5	I am satisfied with family communication about my illness
	GS6	I feel close to my partner (or the person who is my main support)
	GS7	I am satisfied with my sex life
Emotional Well-being	GE1	I feel sad
	GE2	I am satisfied with how I am coping with my illness
	GE3	I am losing hope in the fight against my illness
	GE4	I feel nervous
	GE5	I worry about dying
	GE6	I worry that my condition will get worse
Functional Well-being	GF1	I am able to work (include work at home)
	GF2	My work (include work at home) is fulfilling
	GF3	I am able to enjoy life
	GF4	I have accepted my illness
	GF5	I am sleeping well
	GF6	I am enjoying the things I usually do for fun
	GF7	I am content with the quality of my life right now
Additional Concerns (19-item palliative subscale)	PAL1	I maintain contact with my friends
	PAL2	I have family members who will take on my responsibilities
	PAL3	I feel that my family appreciates me
	PAL4	I feel like a burden to my family
	B1	I have been short of breath
	PAL5	I am constipated
	C2	I am losing weight
	O2	I have been vomiting
	PAL6	I have swelling in parts of my body
	PAL7	My mouth and throat are dry
	Br7	I feel independent
	PAL8	I feel useful
	PAL9	I make each day count
	PAL10	I have peace of mind
	Sp21	I feel hopeful
	PAL12	I am able to make decisions
	L1	My thinking is clear
	PAL13	I have been able to reconcile (make peace) with other people
	PAL14	I am able to openly discuss my concerns with the people closest to me

**Table 2:** Patient (n = 60) Demographics

Age (years)		
n	60	
Mean ± SD	65.6 ± 13.0	
Inter-quartiles	56 - 76	
Median (range)	68 (38 - 88)	
KPS		
n	58	
Mean ± SD	67.6 ± 17.8	
Inter-quartiles	50 - 80	
Median (range)	70 (30 - 100)	
Gender		
Male	37	(61.7%)
Female	23	(38.3%)
Primary cancer site		
Prostate	20	(33.3%)
Breast	11	(18.3%)
Lung	7	(11.7%)
Renal Cell	5	(8.3%)
Oesophagus	3	(5.0%)
Colorectal	2	(3.3%)
Unknown	2	(3.3%)
Others	10	(16.7%)
Clinic		
Radiation Oncology	36	(60.0%)
Medical Oncology	3	(5.0%)
Palliative Care Unit	9	(15.0%)
Others	12	(20.0%)
Patient status		
Outpatient	46	(76.7%)
Inpatient	14	(23.3%)

**Table 3:** Health-Care Professional (n = 56) Demographics

Years of professional experience		
n	56	
Mean ± SD	7.0 ± 6.0	
Inter-quartiles	2 - 10	
Median (range)	6 (1 - 25)	
Gender		
Male	31	(55.4%)
Female	25	(44.6%)
Profession		
Radiation Oncologist	24	(42.9%)
Radiation Therapist	10	(17.9%)
Nurse	6	(10.7%)
General Practitioner in Oncology	2	(3.6%)
Palliative Care Physician	2	(3.6%)
Medical Oncologist/Haematologist	1	(1.8%)
Others	11	(19.6%)

Patients and HCPs both felt items regarding personal and emotional well-being were of greatest importance. Emotional support from family (GS2: 40.9%) was ranked as most important followed by pain (GP4: 38.6%), lack of energy (GP1: 31.8%) and able to enjoy life (GF3: 29.6%) (Table 4). HCPs ranked pain (GP4: 73.2%), lack of energy (GP1: 63.4%), nausea (GP2: 51.2%) and dyspnea (B1: 51.2%). Patients rated nausea at 18.2 % and dyspnea at 9.1%. HCPs tended to rate physical symptoms such as nausea, vomiting and dyspnea much higher than patients. In addition HCPs rated all items as being much more important than patients (top item by HCPs rated to be included by 73%, whereas top item by patients was only 41%).

**Table 4:** Percentage of Patients and HCPs Rating as a Top 10 Item

Order	Item	% from Patients Responses	% from HCPs Responses
1	GP4	38.64%	73.17%
2	GP1	31.82%	63.41%
3	GP2	18.18%	51.22%
4	GE5	22.73%	46.34%
5	GE1	25.00%	43.90%
6	GS2	40.91%	26.83%
7	GF7	25.00%	39.02%
8	PAL4	25.00%	39.02%
9	B1	9.09%	51.22%
10	GE2	20.45%	39.02%
11	GP5	20.45%	34.15%
12	PAL5	25.00%	29.27%
13	GE6	27.27%	21.95%
14	GF3	29.55%	19.51%
15	O2	11.36%	36.59%
16	GF5	13.64%	34.15%
17	PAL14	25.00%	21.95%
18	BR7	15.91%	29.27%
19	GP7	20.45%	24.39%
20	GF4	22.73%	19.51%
21	PAL10	22.73%	19.51%
22	PAL12	15.91%	24.39%
23	L1	NA	19.51%
24	C2	20.45%	17.07%
25	GP3	15.91%	17.07%
26	GS6	18.18%	12.20%
27	PAL2	22.73%	7.32%
28	SP21	27.27%	2.44%
29	GS3	13.64%	14.63%
30	GS4	13.64%	14.63%
31	GP6	13.64%	9.76%
32	PAL7	13.64%	9.76%
33	PAL8	15.91%	7.32%
34	PAL1	11.36%	NA
35	PAL3	11.36%	NA
36	GE4	13.64%	7.32%
37	GF6	13.64%	7.32%
38	GS5	9.09%	7.32%
39	PAL13	9.09%	7.32%
40	GE3	11.36%	4.88%
41	GS7	NA	7.32%
42	PAL9	9.09%	4.88%
43	GS1	11.36%	2.44%
44	GF1	6.82%	4.88%
45	PAL6	6.82%	4.88%
46	GF2	2.27%	4.88%

NA: not available

## Discussion

It is generally accepted that the patients’ perspective is the gold standard for the measurement of health related quality of life and as a result, they should be the primary source regarding what issues are to be included in a health related quality of life (HRQOL) assessment tool [4]. Patients are best able to define and measure their own HRQOL because it is such a subjective experience [5]. In some situations, this may not be possible and a proxy may be asked to rate a patients’ QOL [6]. In general, HCPs tend to outline what is typical in any given situation and therefore provide an external evaluation of the patients’ problems and symptoms. This objective perspective is also important in the development of QOL instruments because patients’ improvements are evaluated based on the clinical parameters.

Our study is consistent with previous studies, in that HCPs value specific QOL concerns differently. HCPs tended to put more emphasis on physical symptoms, whereas patients prioritize psychosocial and global issues. Petersen and colleagues observed the poorest agreements between patients and physicians for social and emotional functioning (0.15 each) with best correlation in nausea/vomiting and constipation (0.54 and 0.60, respectively) [7]. Although patients ranked pain as a priority it was not of the utmost significance. Emotional support from family was the number one priority for patients. The progression of physical distress and disability and the threat of impending mortality with advanced disease may also be a challenge to the sense of self, highlighting the growing dependency on caregivers, Also of note, amongst the top ten relevant issues, patients rated two items of physical concern. All other items were psychosocial domains, whereas this was not the case for HCPs.

Communication is one dimension of the therapeutic patient-physician relationship. This should include comprehensive attention in clinical interactions to patients’ physical and emotional wellbeing, allowing them the opportunity to discuss their goals and their fears, and to feel considered as a whole person. In a study by Detmar and colleagues [8], almost all patients expressed a willingness to discuss the physical and emotional aspects of their disease. However, a quarter of the patients were only willing to discuss emotional functioning at the initiative of their physician. An even greater reluctance was observed concerning the issues of social functioning and family life, with 28-36% of patients waiting for the doctor to first raise the topic and another 20% choosing not to hold a discussion on these issues at all. This suggests that patients may be uncertain about which issues are appropriate to be discussed with their physician. Physicians themselves felt that discussion of the physical aspect of their patient’s health was primarily their responsibility, while a number of physicians indicated that the discussion of psychosocial health problems should be shared with other health care providers. In the case of emotional and social functioning, all physicians indicated that they generally defer the initiation of the topics to their patients.

The importance of screening for psychological disturbances, such as anxiety and depression, in cancer is now recognized as an essential part of comprehensive patient care. Guidelines for distress screening advocate comprehensive assessments of patients' emotional, physical and social or practical needs - all elements that may interfere with the ability to cope effectively with cancer and to participate in treatment [9, 10]. However, screening will only have a positive effect on patient outcomes if it is complemented by a strong institutional commitment to providing adequate treatment resources and longitudinal follow-up [11, 12]. These resources may be most acceptable when they are integrated with routine care, although there is a subset of patients who are reluctant to accept psychosocial care due to stigma, cultural beliefs or unfamiliarity with intervention of this kind. Oncologists play a critical role in normalizing, de-stigmatizing and educating such patients about the importance of psychosocial care.

Limitations of this study are its small sample size and we do not have the adequate sources for evaluation of differences in valuation between HCPs who treat the physical symptoms of cancer pain (oncologists and surgeons) from those who see patients from a broader perspective such as social workers and spiritual support workers. Overall, our study demonstrates a difference in patient and HCPs perspectives on most important issues contributing to quality of life. It is important for HCPs to recognize these differences to better understand the patients’ well-being. For example, it is evident that psychosocial issues may be considered as less important for HCPs but may be a significant component of poorer quality of life for patients.
